# Evolutionary Variability of W-Linked Repetitive Content in Lacertid Lizards

**DOI:** 10.3390/genes11050531

**Published:** 2020-05-11

**Authors:** Grzegorz Suwala, Marie Altmanová, Sofia Mazzoleni, Emmanouela Karameta, Panayiotis Pafilis, Lukáš Kratochvíl, Michail Rovatsos

**Affiliations:** 1 Department of Ecology, Faculty of Science, Charles University, 12844 Prague, Czech Republic; g.t.suwala@gmail.com (G.S.); marie.altmanova@natur.cuni.cz (M.A.); sofia.mazzoleni@natur.cuni.cz (S.M.); lukas.kratochvil@natur.cuni.cz (L.K.); 2 Institute of Animal Physiology and Genetics, The Czech Academy of Sciences, 27721 Liběchov, Czech Republic; 3 Department of Zoology and Marine Biology, Faculty of Biology, University of Athens, 15784 Athens, Greece; emykarameta@biol.uoa.gr (E.K.); ppafil@biol.uoa.gr (P.P.)

**Keywords:** C-banding, evolution, FISH, GATA, heterochromatin, karyotype, microsatellites, sex chromosomes, telomeres

## Abstract

Lacertid lizards are a widely radiated group of squamate reptiles with long-term stable ZZ/ZW sex chromosomes. Despite their family-wide homology of Z-specific gene content, previous cytogenetic studies revealed significant variability in the size, morphology, and heterochromatin distribution of their W chromosome. However, there is little evidence about the accumulation and distribution of repetitive content on lacertid chromosomes, especially on their W chromosome. In order to expand our knowledge of the evolution of sex chromosome repetitive content, we examined the topology of telomeric and microsatellite motifs that tend to often accumulate on the sex chromosomes of reptiles in the karyotypes of 15 species of lacertids by fluorescence in situ hybridization (FISH). The topology of the above-mentioned motifs was compared to the pattern of heterochromatin distribution, as revealed by C-banding. Our results show that the topologies of the examined motifs on the W chromosome do not seem to follow a strong phylogenetic signal, indicating independent and species-specific accumulations. In addition, the degeneration of the W chromosome can also affect the Z chromosome and potentially also other parts of the genome. Our study provides solid evidence that the repetitive content of the degenerated sex chromosomes is one of the most evolutionary dynamic parts of the genome.

## 1. Introduction

Sex chromosomes evolve from a pair of autosomes, after one of them acquires a sex-determining locus [1,2,3]. This locus is on the Y or W chromosome and thus is restricted to a single sex, which affects subsequent processes in the nearby, linked loci. The region around this sex-determining locus progressively stops recombining with their respective homologous regions on the X/Z counterpart, possibly due to inversions [4] or other mechanisms decreasing the frequency of recombination. Over time, the cessation of recombination triggers more structural changes, mainly on the Y and W chromosomes, including the accumulation of deleterious mutations, the degradation of the gene content, the accumulation of repetitive elements, and/or the heterochromatinization. The differentiation process of the X/Z and Y/W chromosomes differs significantly among independently evolved sex determination systems in traits such as the degree of recombination suppression, the heteromorphism of sex chromosomes, and the sharing of gene and repeat content between sex chromosomes.

Sex chromosomes evolved independently in numerous animal and plant lineages, probably mainly to ensure a stable sex ratio in populations and to contribute to the resolution of the conflict between sexes over traits expression via the accumulation of sexually antagonistic alleles [5]. The differentiation of sex chromosomes is a complex and only partially understood process connected to a balance between adaptive and potentially harmful processes. The loss of numerous functional genes from the Y/W chromosomes, the increased frequency of transposons and other repetitive elements in the genomes, heterochromatinization, and the changes in gene expression due to these processes should often have negative fitness effects on the organism. On the other hand, many organismal lineages were able to cope with these potentially detrimental effects associated with sex chromosome differentiation, and differentiated sex chromosomes seem even to act as an “evolutionary trap” [6] in the sense that once evolved, they appear to be very evolutionary stable in the long term. Differentiated sex chromosomes stabilize the sex determination system for dozens of millions of years, as was documented by molecular and cytogenetic evidence for example in anguimorphan lizards, birds, caenophidian snakes, iguanas, lacertids, geckos of the genus *Paroedura*, softshell turtles, and viviparous mammals [7,8,9,10,11,12,13,14,15,16,17,18]. Recent studies in viviparous mammals, birds, iguanas, anoles, monitor lizards, and caenophidian snakes revealed a striking dichotomy: the gene and repetitive content of their Y/W chromosomes differs significantly even between closely related species [19,20,21,22,23,24,25,26,27,28,29,30], despite the long-lasting stability of sex determination systems in these lineages and the extensive between-species homology of their X/Z-specific gene content [7,8,10,12,25]. The contradiction between the similarity of the gene content of the X/Z chromosomes in comparison to the variability of the gene and repetitive content of their Y/W counterparts still remains unresolved. Notably, heterochromatic and/or low-complexity genomic regions such as centromeres and differentiated Y/W sex chromosomes are insufficiently sequenced, assembled, and annotated with the current high-throughput sequencing methodologies and bioinformatic tools [31]. As a result, either the heterogametic sex is often excluded from genome sequencing projects, or the regions from the Y/W chromosomes are poorly assembled and annotated [31]. Therefore, we currently have limited knowledge if the between-species variability of the Y/W gene and repetitive content is exceptional, as research has been restricted among amniotes mainly to a few up to now studied lineages, or whether it is common during sex chromosome differentiation.

Simple repeats, such as mini- and microsatellites, are often overabundant on sex chromosomes [32,33]. Their function is largely unknown. It was speculated that they contribute to the cessation of recombination, formation of heterochromatin, changes in gene expression, or that different kinds of these repeats are accumulated on sex chromosomes randomly, largely reflecting historical contingency [32,34]. The important functional role of such sequences would predict that the pattern of the distribution of their accumulation should be relatively conserved across species of the same lineage. In this context, we selected the lizards of the family Lacertidae to explore the variability of the repetitive content of sex chromosomes between species across a wide phylogenetic scale in another model system. Previous cytogenetic studies demonstrated that all studied lacertids have highly differentiated ZZ/ZW sex chromosomes [34,35,36,37,38,39,40,41,42,43,44]. The majority of chromosomes in lacertids are acrocentric gradually decreasing in size. Therefore, the sex chromosomes cannot be identified by morphology; however, the W chromosome is heterochromatic, visible after C-banding in all studied species [34,36]. The size of the W chromosome varies among lacertid species from small to medium [34,36,45]. In addition, the W chromosome seems to be enriched in satellite motifs in *Acanthodactylus lineomaculatus*, *Eremias velox*, and several species from the genera *Lacerta* and *Timon* [38,40,41,42,43,44]. The Z chromosome is also acrocentric, small to medium in size in *E. velox* [46], and with more than 800 protein-coding genes in *Lacerta agilis* and *Podarcis muralis* [47,48]. Based on qPCR-based methodology applied to 45 species, it was recently revealed that the ZZ/ZW sex chromosomes are homologous across lacertids and that they were highly differentiated already in the common ancestor of the family living approximately 85 million years ago [16,49].

In the current study, we tested the presence of accumulations of selected microsatellite motifs that tend to accumulate on the sex chromosomes of vertebrates by fluorescence in situ hybridization and compared their distribution on the W chromosome of 15 species of lacertids selected for their phylogenetic position. Our aim is to explore the evolutionary dynamics of the accumulation of microsatellite motifs and heterochromatin distribution on the sex chromosomes across the phylogenetic scale of lacertids and to expand our knowledge on the processes of sex chromosome differentiation.

## 2. Materials and Methods

### 2.1. Studied Material

We studied 30 individuals belonging to 15 species of lacertids: *Acanthodactylus schreiberi, Eremias arguta, Gallotia galloti, Gastropholis prasina, Lacerta bilineata, Lacerta media, Lacerta strigata, Lacerta trilineata, Latastia longicaudata, Phoenicolacerta troodica, Podarcis siculus, Takydromus dorsalis, Takydromus sexlineatus, Timon lepidus*, and *Timon tangitanus* (Table S1). Individuals from the species *A. schreiberi* and *Ph. troodica* were collected from the wild in Cyprus (permissions 02.15.007.003.001/04.05.002.005.006 issued from Department of Environment, Ministry of Agriculture, Republic of Cyprus), while individuals from 13 other species were obtained from the pet trade. Blood samples were collected from the vein located at the ventral side of tails with a heparinized syringe. The processing of the biological material was carried out under the supervision and with the approval of the Ethics Committee of the Faculty of Science, Charles University in Prague followed by the Ministry of Education, Youth and Sports of the Czech Republic (permissions No. 15251/2012-30, 35484/2015-14 and 8604/2019-7).

### 2.2. Chromosome Preparations and Staining

Mitotic metaphase chromosome spreads were prepared from whole blood cell cultures following the protocol described by Pokorná et al. [50]. Chromosomal preparations were stained with Giemsa and karyogram reconstruction was used to identify the diploid number (2n) and morphology of chromosomes. To visualize the accumulation of constitutive heterochromatin, we applied C-banding following the protocol of Sumner [51] with modifications described by Pokorná et al. [50]. Giemsa staining, fluorescence in situ hybridization with probe for telomeric or GATA motifs, and C-banding were applied sequentially in the same metaphase in order to unequivocally identify the sex chromosomes and compare the results among the methods.

### 2.3. Fluorescence In Situ Hybridization (FISH) with Probes for Telomeric and Microsatellite Motifs

The distribution of telomeric repeats in the karyotype was examined by fluorescence in situ hybridization with the pan-telomeric peptide nucleic acid (PNA) probe directly labeled with Cy3 fluorochrome (DAKO, Glostrup, Denmark), following the manufacturer’s protocol with a slight modification of longer hybridization time for 1–2 h. Furthermore, we analyzed the pattern of accumulation of microsatellite repeats in lacertid sex chromosomes by FISH using probes for 22 microsatellite motifs: (A)_30_, (C)_30_, (CA)_15_, (CG)_15_, (GA)_15_, (TA)_15_, (CAA)_10_, (CAC)_10_, (CAG)_10_, (CAT)_10_, (CGG)_10_, (GAA)_10_, (GAC)_10_, (GAG)_10_, (TAA)_10_, (TAC)_10_, (AAGG)_8_, (AATC)_8_, (ACGC)_8_, (GACA)_8_, (GATA)_8_, and (TTTC)_8_. The probes were synthesized and 5′-end biotin-labeled by Macrogen (Macrogen, Seoul, South Korea). Microsatellite mapping was performed on metaphase spreads following the protocol used by Rovatsos et al. [52]. The microsatellite signal was amplified and detected using a system of avidin–fluorescein and anti-avidin antibodies (Vector Laboratories, Burlingame, CA, USA) [28,30,52]. Slides were counterstained with 4′,6-diamidino-2-phenylindole (DAPI) and the antifade medium Fluoroshield (Sigma-Aldrich, St. Louis, MO, USA) or Vectashield (Vector Laboratories, Burlingame, CA, USA). The FISH with telomeric probe and the (GATA)_8_ probe were performed in all studied species (Table S1). Other probes were hybridized to only four species (*Gal. galloti*, *Gas. prasina*, *Lac. media* and *Ti. lepidus*) selected with respect to the phylogenetic and karyotype diversity of lacertids.

### 2.4. Microscopy and Image/Data Analyses

We studied at least 10 metaphases from each specimen per method. We used Ikaros karyotyping software (Metasystems, Altlussheim, Germany) to prepare karyograms from Giemsa-stained metaphases of each species. Images were captured using a Provis AX70 fluorescence microscope (Olympus, Tokyo, Japan) equipped with a DP30BW digital camera (Olympus, Tokyo, Japan) or using an Imager Z2 microscope (Zeiss, Oberkochen, Germany) equipped with a CoolCube 1 digital camera (Metasystems, Altlussheim, Germany). Photos of in situ hybridization experiments were superimposed with color and processed with DP Manager imaging software (Olympus, Tokyo, Japan) or an Isis Fluorescence Imaging System (Metasystems, Altlussheim, Germany).

## 3. Results

### 3.1. Karyotype Reconstruction and Heterochromatin Distribution

**Figure 1:**
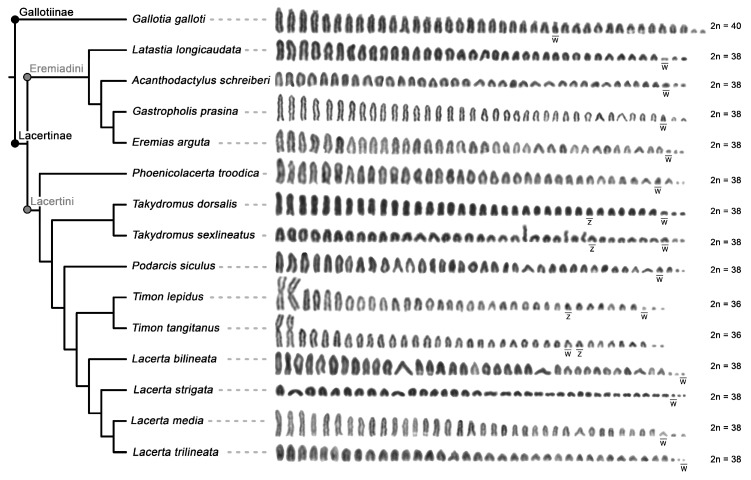
Phylogenetic relationships and Giemsa-stained karyotypes of females in the studied species of the family Lacertidae. The phylogenetic relationships follow Pyron et al. [59] (for an alternative topology see Garcia-Porta et al. [60]). The W chromosomes were identified by C-banding. The Z chromosomes in members of the genera *Timon* and *Takydromus* were detected by fluorescence in situ hybridization (FISH) with a (GATA)_8_ probe.

**Figure 2:**
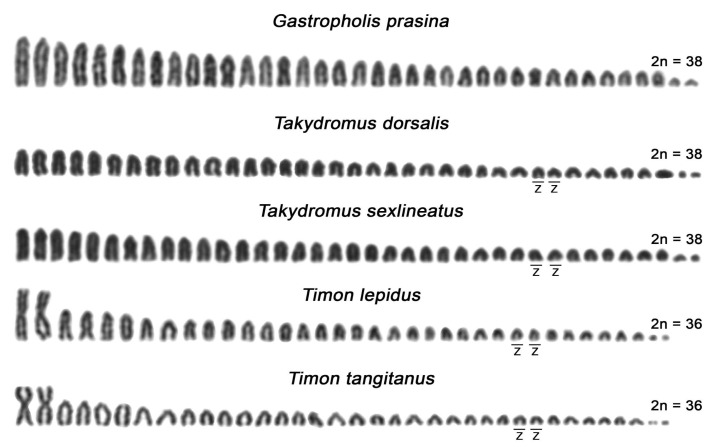
Male karyotypes of previously unstudied species and those with detectable Z chromosomes. The chromosomes were stained by Giemsa. The Z chromosomes of *Timon* and *Takydromus* were detected by FISH with the (GATA)_8_ probe.

**Figure 3:**
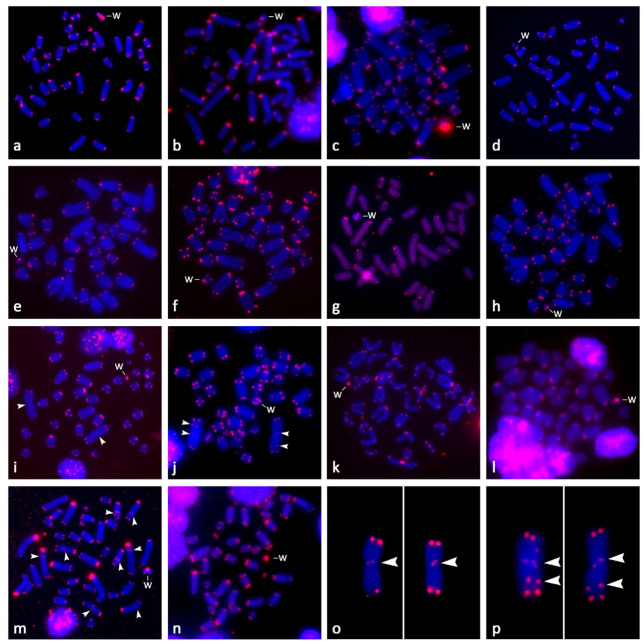
Mitotic metaphase chromosomes hybridized with the telomeric probe in females of (**a**) *Gallotia galloti*, (**b**) *Latastia longicaudata*, (**c**) *Acanthodactylus schreiberi*, (**d**) *Gastropholis prasina*, (**e**) *Eremias arguta*, (**f**) *Phoenicolacerta troodica*, (**g**) *Takydromus dorsalis*, (**h**) *Podarcis siculus*, (**i**) *Timon lepidus*, (**j**) *Timon tangitanus*, (**k**) *Lacerta bilineata*, (**l**) *Lacerta strigata*, (**m**) *Lacerta media*, and (**n**) *Lacerta trilineata*. In *Ti. lepidus* (**o**) and *Ti. tangitanus* (**p**), the largest, metacentric chromosomes were enlarged and the exposure was increased to show the weak signal near the centromere. An additional signal within a chromosome arm is present in *Ti. tangitanus*. Chromosomes were counterstained with DAPI, and the hybridization probes were detected with Cy3 (red). The W chromosomes are indicated; white arrows point at interstitial telomeric repeats.

Karyotypes for the species *A. schreiberi, E. arguta, Gal. galloti, Lac. bilineata, Lac. media, Lac. strigata, Lac. trilineata, Lat. longicaudata, Ph. troodica, Po. siculus, Ta. sexlineatus*, and *Ti. lepidus* agreed with the previous reports [34,35,37,39,53,54,55,56,57,58]. To the best of our knowledge, the karyotypes of *Gas. prasina*, *Ta. dorsalis*, and *Ti. tangitanus* have not been published up to date. Both *Gas. prasina* and *Ta. dorsalis* possess the typical lacertid karyotypes with 2n = 38 chromosomes gradually decreasing in size, with all larger chromosomes acrocentric shared by other lacertids studied here, with the exception of the genera *Timon* and *Gallotia* (female karyotypes are presented in Figure 1, male karyotypes in Figure 2). The karyotype of *Ti. tangitanus* is composed of 2n = 36 chromosomes with the largest pair being metacentric as in the previously studied *Ti. lepidus* [34,35]. The notable difference between karyotypes of the two species from the genus *Timon* can be found only in females. Females of *Ti. lepidus*, but not *Ti. tangitanus*, have 3 microchromosomes that are notably smaller than the other chromosomes. C-banding revealed a strong accumulation of heterochromatin on the smallest macrochromosomes of females in *Ti. tangitanus* and the largest microchromosome of females in *Ti. lepidus*; thus, these chromosomes can be identified as the W chromosomes. *Gal. galloti* has an all-acrocentric karyotype with 2n = 40 chromosomes gradually decreasing in size as previously reported by Cano et al. [57]. In all studied species, the W chromosomes can be identified by C-banding; however, Z chromosomes are difficult to distinguish from autosomes (Figure 1). We were able to identify the Z chromosomes only in four species (both studied species from the genus *Timon* and both species from the genus *Takydromus*; Figure 1 and Figure 2) thanks to their distinct pattern in the FISH experiments with microsatellite probes (Figure 3).

### 3.2. In Situ Hybridization with Telomeric and Microsatellite Repeat Probes

The expected terminal position of the signals with the telomeric probe was observed in all studied lacertids (Figure 3). The hybridization with the telomeric probe was not tested in *Ta. sexlineatus* due to the limited availability of chromosomal material. Centromeric or pericentromeric accumulations of telomeric-like sequences were detectable in acrocentric chromosomes in all tested species with the exceptions of *Ta. dorsalis* and *E. arguta* (Figure 3). Notably, in *Ti. lepidus* and *Ti. tangitanus*, the only metacentric chromosomes (the largest chromosomes in the complement), possess a weak signal in the centromeric region. In *Ta. tangitanus*, additional interstitial telomeric repeats (ITRs) are present in the arm of the metacentric chromosome (Figure 3j,p). The only other species exhibiting ITRs within the chromosomal arms is *Lac. media* (Figure 3m).

**Figure 4:**
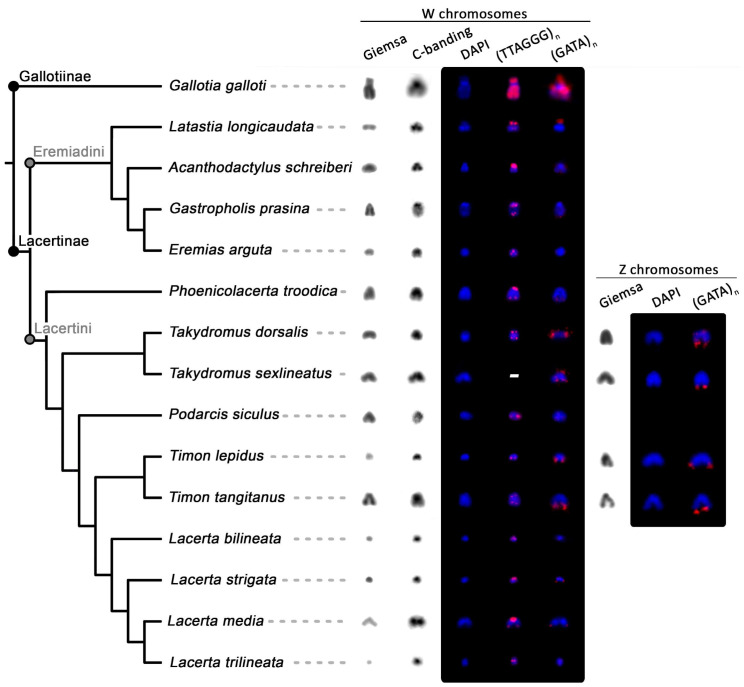
Comparison of morphology, heterochromatinization, and repetitive content (accumulations of telomeric-like repeats (TTAGGG)_n_ and (GATA)_n_ motifs) of the W chromosomes and identified Z chromosomes across the family Lacertidae. In *Ta. sexlineatus*, the telomeric probe was not tested due to the limited availability of chromosomal material. The phylogenetic tree is based on Pyron et al. [59] (for an alternative topology, see Garcia-Porta et al. [60]). Photos of C-banding are inverted. The chromosomes after FISH treatment were counterstained with DAPI (blue), and the probes were detected with fluorescein-avidin D (red).

Regarding accumulations of telomeric-like repeats on the W chromosomes (Figure 3 and Figure 4), among the 15 studied species, *Gal. galloti* presents the most prominent accumulations distributed evenly throughout its W chromosome, excluding the strongly heterochromatic centromere. Other significant accumulations of telomeric-like motifs were found in the pericentromeric region in *A. schreiberi*. The W chromosomes of *Lac. bilineata*, *Lac. strigata*, *Lac. trilineata*, and *Ti. lepidus* had stronger accumulations of terminal telomeric repeats than their autosomes, while autosomes and the W chromosomes do not differ in this respect in *E. arguta*, *Gas. prasina*, *Lac. media*, *Lat. longicaudata*, *Ph. troodica*, *Po. siculus*, *Ta. dorsalis*, and *Ti. tangitanus* (Figure 3 and Figure 4).

**Figure 5:**
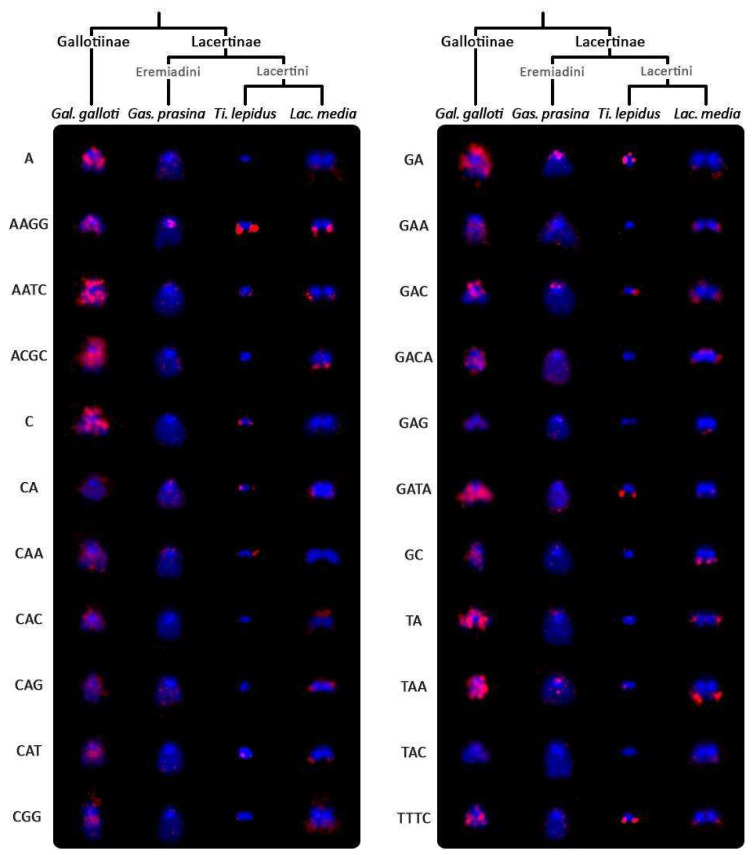
Comparison of the accumulation of 22 microsatellite motifs on the W chromosome of the lacertids: *Gallotia galloti*, *Gastropholis prasina*, *Timon lepidus*, and *Lacerta media*. The chromosomes were counterstained with DAPI (blue), the microsatellite probes were detected with fluorescein–avidin D (red).

The (GATA)_8_ probe hybridized near the centromeric region of the W chromosome in *Lat. longicaudata* and *Gal. galloti*, while it hybridized to telomeric regions in *Lac. strigata*, *Ph. troodica*, *Ta. dorsalis*, *Ta. sexlineatus*, *Ti. lepidus*, and *Ti. tangitanus*. This probe showed no signal on the W chromosomes of *A. schreiberi*, *Gas. prasina*, *Lac. bilineata*, *Lac. media*, *Lac. trilineata* and *Po. siculus* (Figure 4). Additionally, in females of *Ta. dorsalis*, *Ta. sexlineatus*, *Ti. lepidus*, and *Ti. tangitanus*, the (GATA)_8_ probe hybridized also in telomeric regions of an additional small chromosome. We hypothesized that it could be the Z chromosome and tested this hypothesis by the replication of the FISH with (GATA)_8_ probe and C-banding in males of these species (Figure S1). The results supported the hypothesis that the chromosome bearing accumulation of the (GATA)_8_ motif is the Z chromosome: as predicted, two chromosomes possess (GATA)_8_ accumulations in males in all species with the exception of *Ta. sexlineatus*, where a pair of small chromosomes was strongly labeled in both sexes as well (Figure S1c,d). As revealed by C-banding, in contrast to the W chromosomes, the Z chromosomes did not show any accumulations of heterochromatin that would differentiate them from autosomes (results not shown). The only repetitive motif that hybridized to them was the (GATA)_8_ probe (Figure S1). Out of the remaining 21 tested microsatellite probes, only the (AAGG)_8_, (GAC)_10_, and (GA)_15_ motifs showed some degree of accumulation on the W chromosomes in the four species where all motifs were tested (Figure 5). The remaining motifs showed various levels of accumulation on the W chromosome across species, with *Gal. galloti* showing the most extensive accumulations in both the amount and variability of the tested motifs (Figure 5). 

## 4. Discussion

Evidence for ZZ/ZW sex chromosomes based on the copy-number variation of Z-linked genes and/or cytogenetics was available for 72 species of lacertids (recently reviewed by Rovatsos et al. [16]). We have added *Gas. prasina* and *Ta. dorsalis* to this long list covering over 20% of currently recognized lacertid lizards [44]. Nevertheless, the data on the sequence content of the W chromosome and the cytogenetic identification of the Z chromosomes in lacertid karyotypes are scarce. Our FISH experiments with the (GATA)_8_ probe (Figure 4 and Figure S1) revealed that the Z chromosome in four species of lacertids is a small acrocentric chromosome located between the 13th and 16th pair of the complement (Figure 2). Our findings are in accordance with recent cytogenetic evidence in *Eremias velox*, where the Z chromosome was identified as the 13th pair of the complement by the chromosome painting of a W-specific probe hybridized to the lampbrush sex bivalent [46]. Giovannotti et al. [42] based on FISH with a telomeric probe and the C-banding pattern of *Acanthodactylus lineomaculatus* chromosomes identified putative Z chromosomes among the 12th–13th pairs of the complement. In addition, we have estimated from the size of sequencing scaffolds from the genome projects of *Podarcis muralis* and *Lacerta agilis* that the Z chromosomes in these species should be in size between the 13th and 16th pair of the complement [47,48].

In the current study, we confirmed that the W chromosomes across lacertids are quite variable in size as was previously demonstrated for example in birds [61], monitor lizards [62], and snakes [63]. The W chromosomes are tiny in some lacertid species, but small to medium-sized in others (Figure 1). Observations based on the classical cytogenetic techniques led to the suggestion that the variability in the size of the W chromosomes in lacertids reflects the independent emergence of the ZZ/ZW sex chromosomes within this lineage [34]; however, molecular evidence for the homology of ZZ/ZW sex chromosomes across the family [16,64] disproved this hypothesis. As the size of the W chromosome is very different even in closely related lacertid species with otherwise very similar karyotypes (e.g., closely related *Ti. lepidus* and *Ti. tangitanus*, Figure 1), it seems that the size variability in lacertid W chromosomes cannot be attributed to chromosome fissions, fusions, or other significant interchromosomal rearrangements, but it is a result of repeated expansions and contractions of repeat content, as was suggested also for other lineages [26,30,65,66].

Matsubara et al. [40] and Giovannotti et al. [42] identified that the W chromosomes in *Lac. agilis* and in *A. lineomaculatus* are highly enriched in telomeric-like sequences. The accumulation of these sequences in the non-recombining part of the W can be expected, as they are also accumulated on independently evolved sex chromosomes in other squamate lineages [28,38,67]. However, only six out of the 15 here studied lacertid species have accumulations of telomeric and telomeric-like repeats on the W chromosome notably stronger than on autosomes or Z chromosomes (Figure 3 and Figure 4). The strongest accumulation was found in the exceptionally large W chromosome in *Gal. galloti*. The larger accumulation of telomeric-like repeats on the W chromosomes can be considered as an apomorphy of the genus *Lacerta* (Figure 3 and Figure 4), but otherwise, it is difficult to find any clear phylogenetic signal in the pattern. The situation resembles the analogous phylogenetic distribution in caenophidian snakes, where particular lineages exhibit a very diverse extent of the accumulation of telomeric-like repeats on W chromosomes [28]. As telomere shortening is related to aging and numbers of telomeric repeats are an important marker in aging research, we stress that the amount of telomeric-like repeats within chromosomes have to be taken into account during measurements of telomere length. Many techniques for the measurement of telomere size are not able to distinguish between terminal and interstitial positions. Therefore, telomeric-like repeats can give very biased results for the comparison of aging and heredity of telomere length when the variable amount on sex chromosomes is not taken into account [28]. Future studies of telomeres in lacertids have to keep in mind that W chromosomes are highly enriched with telomeric-like repeats in some but not all species of lacertids.

In the past, Banded krait minor satellite DNA repeats (Bkm) consisting of tandem arrays of 26 and 12 copies, respectively, of two tetranucleotides, GATA and GACA repeats, were isolated from caenophidian snakes. Bkm repeats were expected to occur on the heterochromatic sex chromosomes of amniotes, as it was assumed that they play an important role in the emergence of heterochromatin [68,69]. However, it was shown that GATA repeats are notably missing on the heterochromatic W in the lacertid *Eremias velox*, as well as in the heterochromatic regions of the sex chromosomes of several amniote lineages [38,67]. The present results support these findings. The GATA motif is accumulated on some, but not all heterochromatic W chromosomes in lacertids (Figure 4), and it does not co-localize with GACA accumulations on the W across lacertids (Figure 5). Recently, it was shown that the emergence of the accumulations of Bkm repeats during the evolutionary history of caenophidian snakes was not correlated with the emergence of sex-linked heterochromatin, as heterochromatinization likely predeceased the accumulation of GATA-containing repeats on their W chromosomes [70].

The presence and accumulation of the other 21 tested microsatellite repeats varied greatly among four studied lacertid species (Figure 5) as well. *Gal. galloti*, a representative of the basal clade of lacertid phylogeny, had the most prominent accumulation and the biggest W chromosome. On the other hand, the W chromosome of *Gas. prasina* did not present significant accumulations of any of the tested motifs, despite the fact that it has a relatively large W chromosome, too. The variability in the repetitive content of the W chromosomes in lacertid lizards was recently presented also by Giovannotti et al. [43], who showed that the IMO-TaqI satellite DNA repeat accumulates on the W chromosomes of four species from the genus *Lacerta*, but not in *Ti. lepidus*. Future studies based on genomic approaches that enable catalogizing other repeat types in lacertids should test whether the variability in the repetitive content of the W chromosomes is restricted only to microsatellites, or whether the pattern revealed in them is general also for other repeats.

Several authors [38,67] compared repetitive content across independently evolved sex chromosomes and concluded that the identity of the accumulations at least of particular microsatellite sequences on the degenerated sex chromosomes reflect more likely historical contingency rather than a functional aspect of particular repeats. Later studies among reptiles, e.g., in monitor lizards [62,71] and in caenophidian snakes [28,72], documented that the repeat content of degenerated sex chromosomes is highly variable also within a lineage possessing homologous sex chromosomes [29,30,73]. Lacertids can be added to these groups as another example of the high evolutionary dynamics of repetitive content of degenerated chromosomes.

The highly dynamic content of W chromosomes across lacertids contrasts with their otherwise large conservation in karyotypes [45]. In fact, only three different chromosomal numbers occurred among the 15 here studied species (Figure 1), with the most common being the karyotype with 2n = 38 acrocentric chromosomes. The karyotype with 2n = 40 was found also in other members of the subfamily Gallotiinae, which is sister to all other lacertids. It occurs in all species from the genera *Gallotia* and *Psammodromus* [34,35,45,57,58] and might correspond to the ancestral karyotype of the subfamily. Among the studied lacertid species, metacentric chromosomes are present only in the genus *Timon*. The karyotype with 2n = 36 derived from the ancestral 2n = 38 via a Robertsonian fusion [39], and it seems to be a synapomorphy of this genus, as it is present also in *Ti. princeps* [74] and *Ti. pater* [75]. ITRs used to be considered as a marker of chromosomal rearrangements, although there are more mechanisms responsible for their emergence [76]. A previous study by Rojo et al. [39] did not detect any ITRs as a remnant of the fusion in the metacentric chromosome in *Ti. lepidus*. However, we noticed that there is a weak telomeric-like signal corresponding to ITRs in the assumed fusion point, i.e., in the centromeric region in both *Ti. lepidus* (Figure 3o) and *Ti. tangitanus* (Figure 3p). There are also additional ITRs in the chromosome arm of the same metacentric chromosome in *Ti. tangitanus* (Figure 3p). In addition to *Ti. lepidus* and *Ti. tangitanus*, ITRs within chromosomal arms were detected in *Lac. media* (Figure 3m). There was speculation that the origin of ITRs within chromosome arms, a relatively common trait in squamates, can be connected to intrachromosomal rearrangements (e.g., inversions) [73]. This hypothesis should be tested in lacertids when well-assembled genomes enabling the detailed detection of inversions will be available.

In contrast to the heterochromatic W chromosomes, the Z chromosomes were cytogenetically unequivocally distinguished from autosomes in only a few lacertid species [39,46,77] thanks to chromosome-specific hybridization probes or specific DNA methylation patterns. Thus, an interesting observation is that we were able to identify Z chromosomes in four species of two genera. In them, not only W, but also Z chromosomes possess notable accumulations of GATA repeats. With the exception of a pair of small chromosomes in *Ta. sexlineatus*, which can be an evolutionary novelty of this species, the accumulations of these motifs do not accumulate on autosomes, but rather only on the sex chromosomes. We speculate that the Z chromosomes (and in *Ta. sexlineatus* maybe also a pair of autosomes) became “infected” by the repeats from the degenerated W chromosomes. Although largely different in sequences including gene content [64], the lacertid Z and W chromosomes make a bivalent during female meiosis and have pseudoautosomal regions [46]. We assume that a genomic region with GATA repeats was transferred from the W to the Z through recombination and in the case of *Ta. sexlineatus* to autosomes via translocation. These cases suggest that the degeneration process of the W/Y chromosomes might also affect the Z and X chromosomes and potentially also other parts of genomes, which is a phenomenon deserving further study.

## Supplementary Materials

The following are available online at https://www.mdpi.com/2073-4425/11/5/531/s1, Table S1. List of individuals per species and sex analyzed in this study. Figure S1. Mitotic metaphase chromosomes hybridized with the (GATA)_8_ probe in (a) female and (b) male of *Takydromus dorsalis* (TADO), (c) female and (d) male of *Takydromus sexlineatus* (TASE), (e) female and (f) male of *Timon lepidus* (TILE), (g) female and (h) male of *Timon tangitanus* (TITA).
