# Supplementary material for: Evolutionary Variability of W-Linked Repetitive Content in Lacertid Lizards
Source: Genes (Basel). 2020 May 11;11(5):531. doi: 10.3390/genes11050531 (PMC7290949; doi:10.3390/genes11050531)
Supplement: Supplementary file 1 [file genes-11-00531-s001.pdf]

Supllementary Materials.

Table S1. List of individuals per species and sex analyzed in this study.

Species	No of specimens	
	♂	♀
Acanthodactylus schreiberi	-	1
Eremias arguta	-	1
Gallotia galloti	1	2
Gastropholis prasina	2	3
Lacerta bilineata	-	1
Lacerta media	-	1
Lacerta strigata	-	1
Lacerta trilineata	2	1
Latastia longicaudata	-	1
Phoenicolacerta troodica	-	1
Podarcis siculus	-	1
Takydromus dorsalis	1	1
Takydromus sexlineatus	1	1
Timon lepidus	2	2
Timon tangitanus	1	1

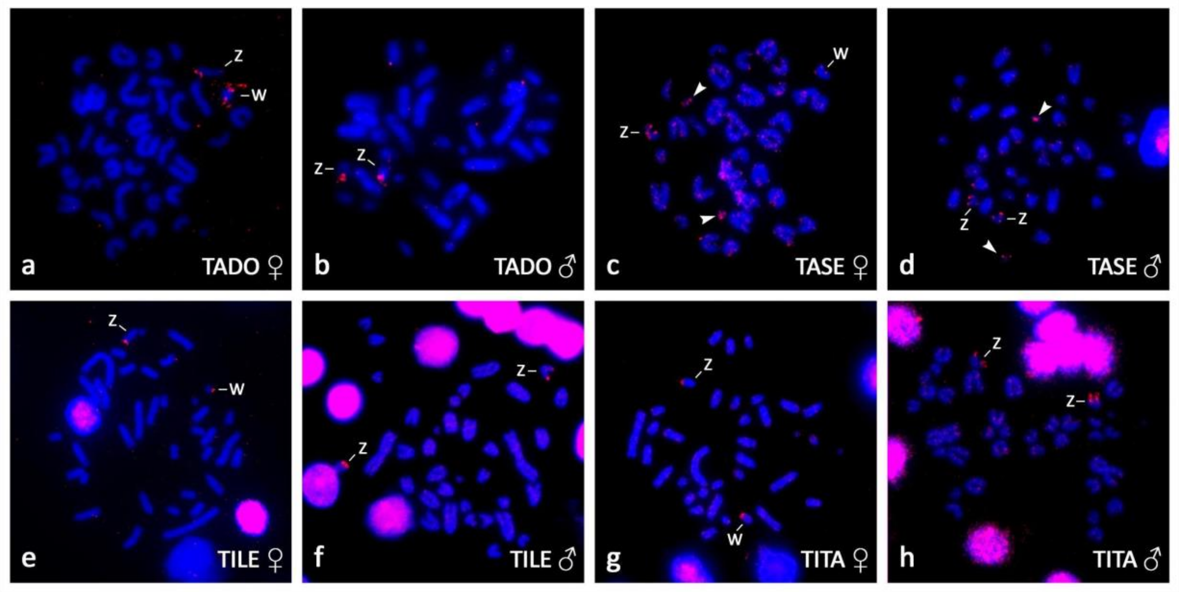

Figure S1. Mitotic metaphase chromosomes hybridized with the $(GATA)_8$ probe in (**a**) female and (**b**) male of *Takydromus dorsalis* (TADO), (**c**) female and (**d**) male of *Takydromus sexlineatus* (TASE), (**e**) female and (**f**) male of *Timon lepidus* (TILE), (**g**) female and (**h**) male of *Timon tangitanus* (TITA). Chromosomes were counterstained with DAPI (blue), the hybridization probes were detected with fluorescein-avidin D (red). The Z and W chromosomes are indicated, microchromosomes in *Takydromus sexlineatus* with additional signals are pointed with white arrows.
